# T-Cell Mechanobiology: Force Sensation, Potentiation, and Translation

**DOI:** 10.3389/fphy.2019.00045

**Published:** 2019-04-02

**Authors:** Devin L. Harrison, Yun Fang, Jun Huang

**Affiliations:** 1 The Graduate Program in Biophysical Sciences, The University of Chicago, Chicago, IL, United States; 2 Section of Pulmonary and Critical Care, Department of Medicine, The University of Chicago, Chicago, IL, United States; 3 Institute for Molecular Engineering, The University of Chicago, Chicago, IL, United States

**Keywords:** T cell, force, mechanotransduction, receptor-ligand interaction, T-cell recognition, T-cell activation, T-cell differentiation, metabolism

## Abstract

A T cell is a sensitive self-referential mechanical sensor. Mechanical forces influence the recognition, activation, differentiation, and function throughout the lifetime of a T cell. T cells constantly perceive and respond to physical stimuli through their surface receptors, cytoskeleton, and subcellular structures. Surface receptors receive physical cues in the form of forces generated through receptor-ligand binding events, which are dynamically regulated by contact tension, shear stress, and substrate rigidity. The resulting mechanotransduction not only influences T-cell recognition and signaling but also possibly modulates cell metabolism and gene expression. Moreover, forces also dynamically regulate the deformation, organization, and translocation of cytoskeleton and subcellular structures, leading to changes in T-cell mobility, migration, and infiltration. However, the roles and mechanisms of how mechanical forces modulate T-cell recognition, signaling, metabolism, and gene expression, are largely unknown and underappreciated. Here, we review recent technological and scientific advances in T-cell mechanobiology, discuss possible roles and mechanisms of T-cell mechanotransduction, and propose new research directions of this emerging field in health and disease.

## INTRODUCTION

T lymphocytes or T cells are white blood cells that play a central role in the adaptive immune system, where the body adapts specificity to foreign antigens. T cells are derived from hematopoietic stem cells and mature in the thymus, thus named T cells. Most T cells in the thymus express αβ T-cell receptors (TCRs) and approximately 5% bear the γ^δ^ TCRs. αβ T cells have been extensively studied while γ^δ^ T cells are much less known. This review will focus on αβ T cells which can be generally divided into two major populations based on their co-receptors and immune functions: CD4^+^ helper T cells and CD8^+^ cytotoxic T cells. CD4^+^ helper T cells orchestrate the full panoply of immune responses by releasing cytokines and chemokines to help other immune cells while CD8^+^ cytotoxic T cells can directly kill pathogen-infected cells [1–3]. Due to the essential role of T cells in the adaptive immune system, they have been extensively studied in immunology. In recent years, the development of T-cell based cancer immunotherapy has shifted the paradigm of cancer treatment. Both checkpoint inhibitors and chimeric antigen receptors (CAR) T cells have been approved by FDA for cancer therapy and showed unprecedented success in clinics [4–9]. Throughout the lifetime and phenotypical trajectory of T cells, they constantly evolve themselves and execute their immune functions in different biochemical and biomechanical coupled environments. The role of biochemical cues in regulating T cells have been extensively studied in the context of T-cell development, migration, activation, differentiation, and function. However, the role of mechanical force, despite its critical importance, is much less investigated, understood, and appreciated. In this paper, we will review some recent advances in T-cell mechanobiology with focuses on T-cell recognition, signaling, metabolism, and genetics, discuss possible key questions and challenges in T-cell selection, differentiation, metabolism, and fate decision and propose new biomechanical approaches to tackle these important problems.

## FORCE IN T-CELL RECOGNITION

**FIGURE 1 |:**
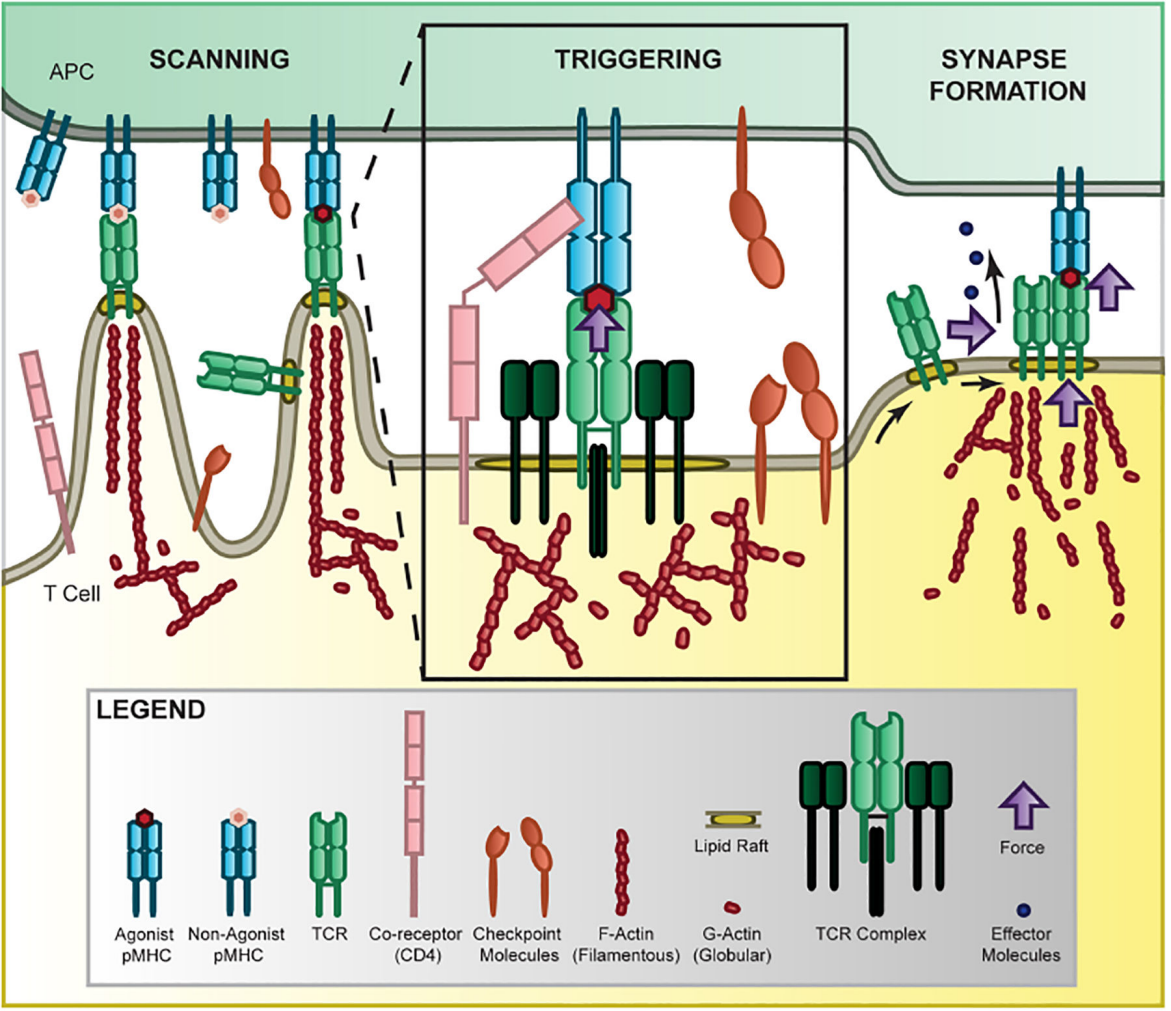
Force in T-cell recognition. T cells scan APCs where they are able to sensitively discriminate activation cues from a large pool of non-activation cues by the TCR binding to MHC presenting self or non-self ligands [10–14]. The 2D agonist pMHC/TCR interaction is further mediated by co-receptor binding [19–22], checkpoint inhibition [23–25], and force through a catch-bond mechanism [11, 26–28]. Once sufficiently triggered, the TCR complexes accumulate together, sliding along the membrane in lipid rafts where they form microclusters and eventually the immunological synapse to localize internal signaling and release of effector molecules [14, 29–33]. This process is mediated by force through the cytoskeleton [34] where polymerizing actin provides tension in the microvilli that scan APC surfaces [35], at the membrane to stabilize the receptor and facilitate receptor sliding [36], and at the synapse to promote adhesion and release of effector molecules.

T cells patrol the body, using their TCRs to search for foreign antigens presented on antigen-presenting cells (APCs) and trigger antigen-specific immune responses, a process called antigen recognition. T-cell antigen recognition is essential to cell-mediated adaptive immune responses. It has been found that T cells can specifically [10, 11] and sensitively [12–14] detect a small number of antigenic peptide-bound major histocompatibility complexes (pMHCs) in the ocean of endogenous ligands displayed on the surface of professional APCs, including B cells [12–17] and dendritic cells [18]. How T cells achieve this sensitivity to antigenic but not endogenous ligands is still enigmatic, but mechanical forces can critically regulate the entire process of T-cell recognition (Figure 1). It has been observed that T cells migrate to the infection sites, deform their shapes, interact with their target APCs, survey their antigens, reorient their cell organelles, and release cytokines or cytotoxins to mediate the antigen-specific adaptive immune responses. All these steps require and involve mechanical forces, which are largely understudied and/or under appreciated. T-cell recognition is the first and essential step for initiating T-cell signaling and activation. Here we review the challenges in fully understanding T-cell recognition, examine current knowledge in T-cell recognition, and discuss how mechanical forces regulate T-cell recognition at the molecular and cellular levels.

### Challenges in T-Cell Recognition

There is considerable controversy about the molecular mechanism of antigen recognition by T cells, which is a complex process by which TCRs bind to pMHCs displayed on APCs’ surface, propagate surface binding across the plasma membrane, trigger T-cell signaling, and ultimately influence immune function. Mechanical forces are involved throughout each step of the recognition process, including cell trafficking, antigen survey, cell adhesion, cytoskeleton reorientation, transmembrane signaling, and cytokine release or cell killing. The spatial scale ranges from a single molecule (~10 nm) to a single cell (~10 μm), and the temporal scale spans from molecular interactions (~ms) to cellular immune responses (~hr). T-cell recognition has the following important characteristics: (1) it is very sensitive—TCRs can recognize even a single foreign pMHC in the presence of abundant self-pMHCs, (2) it is very specific—TCRs can discriminate between closely related amino acids, and (3) it is subjected to substantial mechanical forces. The complexity reflects the uniquely demanding nature of T-cell recognition, which requires the detection of a weak “signal” (very rare foreign pMHCs) in the presence of considerable “noise” (abundant self-pMHCs) at the surface of the cell being surveyed, and precise propagation of such a weak recognition signal across the cell membrane via mechanical force. Many models have been proposed based on the structure, thermodynamics, kinetics, and signaling, but the molecular mechanism of T-cell recognition remains elusive [37–41]. One of the major caveats is that most of these existing models neglect the importance of mechanical forces. In addition, most state-of-the-art mechanical methods utilize artificial or surrogate APCs to present antigens to T cells [11, 19, 42, 43]. It is important to note that future studies should consider using real and physiological relevant APCs such as dendritic cells, B cells and macrophages to study T-cell recognition, as the cellular environment and type of APC could significantly affect the force, signaling, and function of T cells. To fully explain the entire process of T-cell recognition including signal reception, signal transduction, and cellular response, one needs to measure the binding-signaling coupled mechanotransduction of TCRs with enough spatiotemporal resolution under physiological conditions.

### The Mechanosensor TCR

#### TCR-CD3 Complex and TCR Diversity

The cell surface TCR-CD3 complex serves as the unique mechanosensor for T-cell recognition and signaling. Structurally, an extracellular TCR *α*β domain is noncovalently associated with a multisubunit CD3 signaling apparatus, consisting of one CD3εγ heterodimer, one CD3εδ heterodimer, and one CD3ζζ homodimer, which collectively form the TCR–CD3 complex. The CD3εγ and CD3εδ subunits each consist of a single extracellular Ig domain and a single immunoreceptor tyrosine-based activation motif (ITAM), whereas CD3ζ has a short extracellular domain and three ITAMs [44–47]. It is generally thought that the cell surface TCR binds to the pMHC and the CD3 initiates T-cell signaling through phosphorylation. However, far less is known about how TCRs relay the binding of antigens to initiate the CD3 intracellular signaling. Although mechanical force is generally believed to be one of the critical factors in mediating this signaling propagation process, the molecular details remain unclear. Possible solution requires simultaneous examination of TCR-pMHC binding, TCR-CD3 conformational dynamics, and CD3 phosphorylation with high spatiotemporal resolution.

To combat the enormously diverse types of antigens in infections and cancer, the human body needs to generate enough TCR clonotypes with high antigen specificity. Genetically, the diversity of the TCR repertoire is generated by V(D)J recombination in the thymus in a nearly random fashion that rearranges of variable (V), joining (J), and in some cases, diversity (D) gene segments [37]. After recombination, random insertion, deletion, and substitution, a small set of TCR genes can theoretically generate 10^15^ to 10^20^ different TCR clonotypes [48]. Although the actual diversity of a person’s TCR repertoire is much less [49] and difficult to accurately estimate [48], this process results in amino acid sequences in the antigen-binding regions of TCRs that allow for the recognition of antigens from nearly all foreign pathogens as well as mutated self-antigens as seen in cancer [50]. Because T cells stimulate both humoral and cellular immune responses, it is crucially important that T cells only react with foreign antigens while ignoring the large excess of self-peptides. It remains enigmatic how T cells maintain such a diverse TCR repertoire with high specificity to foreign antigens. TCR-pMHC binding kinetics and affinity have been used to explain the molecular mechanism of TCR antigen recognition. So far, most studies measured the kinetic parameters using purified TCRs and pMHCs in solution in which both proteins can diffuse three dimensionally, and therefore termed as 3D binding kinetics and affinity [38]. However, in the physiological conditions, both TCRs and pMHCs are anchored on the live cell membrane, where their orientation, diffusion, association, and dissociation are occurring at the two-dimensional cellular environment subjected to mechanical forces [51], governing the signaling, activation, function, and survival of T cells.

#### Two-Dimensional TCR Binding Kinetics

Different from molecular interactions between antibody and antigen or cytokine and its receptor, where at least one molecule is in solution and can freely diffuse in a three-dimensional (3D) environment, the antigen recognition by T cells is mediated by molecular interactions occurring at the two-dimensional (2D) immunological synapse formed between a T cell and an APC [17, 52], depicted in Figure 1. Most studies have used surface plasmon resonance, a sensitive technique to measure molecular interactions using purified receptors and ligands in a 3D fluid phase [53–56]. Some other studies used pMHC tetramers to study the binding kinetics [57–59]. Although these *in vitro* 3D measurements nevertheless provide the basic kinetic information of TCR-pMHC interactions, they cannot faithfully reveal the physiological binding properties of TCRs *in situ*, as these measurements removed the molecules from their cellular environment, where they initiate signaling and function. Most 2D TCR-pMHC measurements at cell membrane are significantly different from 3D TCR–pMHC binding in solution. These differences have prompted us to re-examine our current views of TCR recognition.

The kinetic differences between 2D and 3D TCR-pMHC interactions come from both the reaction dimension and the complexity of the cellular microenvironment. Firstly, these two interactions are geometrically different. For 3D interactions, at least one molecule is in solution that can diffuse three dimensionally without the constraints of the cell to encounter its counterpart molecule for binding. While for 2D interactions, both the receptors and ligands are surface-bound and only can diffuse at the cell membrane. In order to have 2D interactions, two cells must first make contact so that the surface receptors and ligands can encounter and interact with each other. The dimensional differences result in different units for binding affinity *K*_a_ (molecular concentration, M^−1^ for 3D and molecular density, m^2^ for 2D) and on-rate *k*_on_ for 2D or 3D interactions, although they have the same unit of off-rate *k*_off_ (time, s^−1^). The second difference is from the complex cellular environment in which the molecules reside. Surface molecules are often linked to cytoskeleton, sitting on the lipid bilayer of the cell membrane, or associated with intracellular signaling molecules. It has been found that the molecular length, dimension and orientation, cell surface roughness, membrane nanostructure, cytoskeleton polymerization, lipid composition, and molecular/cellular signaling can profoundly impact 2D receptor-ligand binding kinetics [11, 19, 29–31, 60–66]. Several 2D techniques have been used to measure the molecular interactions at live T-cell membranes with pMHCs presented by surrogate APCs or supported planar lipid bilayers. These techniques can be generally classified as mechanical-based and fluorescence-based methods. The pros and cons of some of the 2D techniques have been summarized in a previous review paper [67]. Here we will review some representative papers and summarize their results.

There are several major fluorescence-based 2D kinetic measurements, including a single-molecule fluorescence resonance energy transfer (smFRET) [20], a single-molecule diffusion assay [68], a single-molecule TCR-pMHC-ZAP70 tracking assay [69], and a DNA-based TCR imaging method [70]. Here we will mainly focus on the smFRET assay to highlight the uniqueness and importance of measuring 2D receptor-ligand interactions in the physiological cellular microenvironment [20]. To use smFRET to measure the kinetics of TCR–pMHC binding *in situ*, individual TCRs on the live primary T-cell surface and single pMHCs embedded in the planar lipid bilayer are, respectively, labeled with a pair of FRET donor and acceptor fluorophores. The TCR-pMHC interaction brings the donor and acceptor into close proximity and trigger smFRET. The off-rate is derived by monitoring the duration of smFRET signals during synapse formation after photobleaching correction. The 2D binding affinity is estimated by measuring the concentrations of free TCRs, free pMHCs and TCR-pMHC complexes in the 2D immunological synapse as well as individual TCR microclusters. Compared to 3D measurements, the 2D on-rate and off-rate increased ~100-fold and ~10-fold, respectively. Molecular orientation and clustering of TCRs have been suggested by the authors to be the causes of 2D and 3D kinetic differences. In a following study, Klein used a same FRET system to measure the 2D off-rate of 5C.C7 TCR in bulk [71]. However, his bulk FRET results (*k*_off_ = 0.17 s^−1^) are significantly different from those obtained by smFRET (*k*_off_ = 0.41–6.36 s^−1^) [20]. Such differences were further revealed by a study from the Groves group, who used a single-molecule tracking 2D assay to measure the dwelling time of 5C.C7 TCR and found a value of 5.2 s (*k*_off_ = 0.19 s^−1^) [69]. Although it is not clear which factors caused such a discrepancy among different studies, more work need to be done to fully address this issue, which is of critical importance in understanding TCR recognition.

Simultaneously, we used two mechanical methods, the micropipette adhesion frequency assay and the thermal fluctuation assay, to measure the TCR–pMHC interactions at the live T-cell membrane [11]. Both mechanical methods, described in detail later in section Methods to Study Force-Mediated Receptor-Ligand Interactions, used a human red blood cell (RBC) as sensitive force sensor to detect single TCR-pMHC bond with piconewton sensitivity. Unexpectedly, the 2D off-rates are up to 8,300-fold faster than the 3D off-rate. Although we cannot directly compare on-rate and affinity between 2D and 3D measurements because they have different units in this case, we have found that 2D affinities and on-rates of the TCR for a panel of pMHC ligands possess far broader dynamic ranges and they are excellent predictors of T-cell responses [11]. Please note that the kinetics and affinities measured here were in the absence of external force (or zero force). In the following sections, we will discuss how force regulates TCR-pMHC binding kinetics and bond lifetime. Our two biomechanical assays consistently show fast kinetics in the 2D cellular environment. Also, disruption of cytoskeleton and lipid rafts by pharmacological agents dramatically changed the 2D binding kinetics and affinity in our biomechanical assays, highlighting the importance of cellular environment to the TCR-pMHC interaction *in situ*.

There are major differences between fluorescent assays and biomechanical methods [51]. The mechanical methods probe the formation of a small number of bonds within a few seconds of transient cell contacts, while fluorescent assays measure interactions over minutes of continuous cell contacts at the ordered structure of immunological synapse (although the measurements are at the single-molecule level). Another difference is that fluorescent assays measure the TCR-pMHC interactions in the presence of adhesion molecules such as B7 and ICAM-1, while mechanical methods purely measure the TCR-pMHC interaction without other interactions. The presence of other receptor-ligand interactions in an ordered structure could greatly affect TCR ligand binding. Also, directly comparing 2D affinities and on-rates between fluorescent assays and mechanical methods are not established, one of the difficulties being precise measurements of the effective contact areas in both assays.

#### Force Measurements of TCR/pMHC Interaction

During T-cell recognition, T cells actively migrate to the infection sites, contact with target cells, scan target cell surface antigens, form immunological synapses, and initiate immune responses (Figure 1). T cells are subject to external forces as well as generate their own internal forces. The external forces include shear stress, compression, and tension in the circulation system while internal forces are generated by cell adhesion, membrane tension, and actomyosin cytoskeleton contraction, depicted in Figure 1. Several studies have suggested that the TCR is a mechanosensor [72–74]. As TCR/pMHC interaction is the key molecular event that initiates and governs the T-cell immune response, we need to fully understand how mechanical forces regulate TCR recognition in a physiological setting. It has been shown that mechanical forces trigger T-cell signaling via TCR/pMHC interactions but not other receptor-ligand interactions on the T-cell surface [75]. Also, the forces mediating T cell/dendritic cell interactions are peptide-dependent and match the potencies of the peptide in activating T-cells [18], shown as force controlled calcium signaling and IL-2 secretion. In addition, mechanical forces generated from receptor sliding or movement during clustering as well as from actin flow, drive TCR-dependent recognition and signaling [76, 77]. It has been shown that mechanical force regulates the TCR/pMHC binding kinetics [78] as well as increases the T-cell recognition sensitivity by a magnitude of ~100-fold [79]. Recently, a type of bond that changes its kinetics in response to force, named catch bond, has been identified as an important mechanism for antigen discrimination by the mechanosensor TCR [26, 27, 78], and we will discuss this in details below.

#### TCR-pMHC Catch Bond

A catch bond is a noncovalent bond whose lifetime increases with tensile force applied to the bond. Catch bond is counterintuitive because bond lifetimes are expected to decrease with force, termed as slip bond [80]. Catch bond was initially proposed by Dembo et al. [81] and was first decisively observed in selectinligand interactions by Zhu and colleagues in 2003 [82]. Since then, catch bond have been found in many other receptor-ligand interactions [83–88]. Recently, Zhu and colleagues further found that TCR-pMHC interaction forms catch bond [26, 78]. Using the biomembrane force probe (BFP, described in section Methods to Study Force-Mediated Receptor-Ligand Interactions) and CD8^+^ OT-I transgenic TCR system, Zhu and colleagues found that force regulates bond lifetimes and activation levels in a peptide-specific manner. Importantly, catch bond was found for agonists while slip bond was found for antagonists in both CD4^+^ and CD8^+^ T-cell systems. The peak lifetimes of catch bond and slip bond are at ~10 pN and 0 pN, respectively. This provides an explanation for the puzzling negative correlation between peptide potencies and off-rates at zero force, because it is hard to directly explain the observation that the off-rate of an agonist is faster than those of weak ligands [11, 78]. When the force reaches ~10 pN, the negative correlation is reversed to positive correlation by the catch bond mechanism, shown as that the agonist has the longest lifetime (slowest off-rate) compared to other weak ligands [26]. The catch bond mechanism reconciles the appearing contradiction of TCR dwelling time [11] and the kinetic proofreading model [28, 89–91]. In addition, it has been found that both the magnitude and duration, i.e., the accumulation of force are important for the activation of the T-cells. In summary, force reinforces antigen discrimination between closely related peptides through this catch-slip mechanism. Mechanistically, the fast off-rate at zero force allows fast scanning of antigens by TCRs. Following binding, forces generated from the cellular environment drive the formation of catch bonds to stabilize the TCR-pMHC interaction of agonist but not the weak ligands, thereby selectively prolonging the cumulative bond lifetime of agonist ligands to trigger antigen-specific activation of T-cells.

The TCR-pMHC catch bond was also found using optical tweezers, a method described later in section Methods to Study Force-Mediated Receptor-Ligand Interactions [27]. This work shows that TCR is a mechanosensor that can be activated by force upon pMHC ligation. The authors discovered a catch-and-release TCR structural conversion correlating with ligand potency wherein a strongly binding/compact state transitions to a weakly binding/extended state. They proposed an allosteric mechanism that the CbFG loop region allosterically controls the V domain module’s catch bond lifetime and peptide discrimination via force-driven conformational transitions.

Catch bond also can be used to examine whether a TCR-pMHC interaction is productive. We previously found the 2D TCR-pMHC binding affinity correlates with TCR activation using a panel of pMHC ligands [11]. However, Garcia et al. found that high frequency of human TCRs are refractory to activation by pMHC ligands with high binding affinity [28]. Analysis of 3D affinity, 2D dwell time, and crystal structures of stimulatory vs. non-stimulatory TCR-pMHC interactions failed to explain their differences in signaling outcome. Recently, they found that TCRs use the catch bond mechanism to differentiate stimulatory from non-stimulatory interactions with same robust binding. Therefore, force-dependent catch bonds may serve as a checkpoint in governing TCR immune responses.

### Co-receptor CD4/8 and PD-1 Molecule

In addition to TCRs, T-cell recognition also involves many other surface receptors. Here we will focus on co-receptor CD4/CD8 and programmed cell death protein 1 (PD-1). The co-receptor CD4/CD8 plays a critical role in T-cell signaling. It is generally thought that co-receptor CD4/CD8 binding to MHC brings Lck to mediate the phosphorylation of CD3 and ZAP-70. It has been found that blocking co-receptor CD4/CD8 significantly reduces T-cell signaling by more than 100-fold [15, 20, 92–95]. However, co-receptors CD4 and CD8 bind to MHC ligands with very low 3D affinities (*K*_D_, ~100 μM) [21, 96–98]. Consistently, 2D CD8-MHC interaction has a very low 2D affinity [99] and 2D CD4-MHC binding is too weak to precisely quantify by current techniques [78]. Clearly, there is a significant discrepancy between the strong signaling and weak binding of co-receptor CD4/CD8. In addition, there might be differences between CD4 and CD8 as well. It has been previously found that CD8 greatly promotes TCR recognition in 2D binding [19, 22]. In sharp contrast, CD4 does not appear to affect the both the 2D and 3D kinetics of TCR–pMHC interactions [20, 21]. We speculate that forces might play an important role in mediating co-receptor CD4/CD8 binding and signaling. To our limited knowledge, we did not find any conclusive force experiments that quantitatively measure how forces regulate the interactions between co-receptor CD4/CD8 and MHC ligand. However, recent studies show CD8 participates in a trimolecular catch bond with TCR-pMHC in a peptide specific manner, beginning to address the link between 2D affinity, force, and CD8 co-receptors, postulating a possible mechanism for thymocyte selection [42, 100]. Such force measurements will be critical to elucidate the major discrepancy between signaling and binding of co-receptors as well as the differences between CD4 and CD8.

PD-1, one of the checkpoint and key molecules for immunotherapy [101–103], has been extensively studied biochemically, although its detailed mechanisms regarding modulation of T-cell immune responses are still not fully understood. It has been found that PD-1 mediated inhibition is through both CD28 [104, 105] and the TCR complex [23–25], with CD28 as the primary target. Interestingly, PD-1 mediates not only trans PD-1/PD-L1 interactions between the T cell and the target cell, but also cis PD-1/PD-L1 and PD-L1/B7–1 interactions at the same cell surface [106, 107]. The co-existing trans- and cis-interactions suggest a much more complex regulation network of T-cell activation and inhibition. Zhu and colleagues have measured 2D binding kinetics for PD-1 and ligand interactions [43]. However, no force measurements have been performed for PD-1 yet. It is highly possible that, similar to the TCR or integrin, forces could regulate the binding and signaling of PD-1-mediated inhibition. Therefore, further mechanotransduction studies should include PD-1 as an important research focus. Such investigations will not only advance our understanding of the inhibitory signaling pathway of T cells, but also can guide the rational design of immunotherapy for effective cancer treatment.

### Cytoskeleton, Lipid and Receptor Nanostructure

The cytoskeleton is a highly dynamic network of filamentous proteins that exists in the three-dimensional space to connect and integrate different regions and components of a T cell. Upon TCR-pMHC ligation, T cells undergo a series of complex cytoskeleton-dependent, force-mediated activities, including cell adhesion [108–110], receptor clustering [29–31], cellular polarization [111], actin depletion [112], CD45 exclusion [113], synapse formation [17, 114], signaling [34, 79, 115, 116], and immune functions [112, 117, 118] (Figure 1). The cytoskeleton provides the dynamic cellular framework to orchestrate these processes to ultimately control T-cell signaling [111]. We have previously found that disruption of the cytoskeleton dramatically reduced 2D TCR-pMHC binding [11] and 3D pMHC dodecamer staining [119]. Evidently, T cells generate and apply mechanical forces to regulate molecular and cellular events through their cytoskeleton. Accumulating evidence further supports the importance of force in T-cell signaling and function that directly involves cytoskeleton regulations [79, 116, 117], including a recent study showing that forces generated by T cells are regulated by dynamic microtubules at the interface [120]. Actin polymerization and turnover are energy dependent and essential for force generation by T cells. Actin polymerization requires ATP and some metabolic enzymes have been shown to be dynamically regulated by the cytoskeleton [121–124]. To fully elucidate the T-cell recognition mechanism, the role of forces generated and transduced by the cytoskeleton during antigen recognition needs to be fully addressed and understood at the molecular and cellular levels: one needs to understand how the multifunctional cytoskeleton provides active transportation of TCRs and other surface molecules, how the cytoskeleton uses energy to generate and transduce mechanical force, how the cytoskeleton works as an efficient machine to transduce signals in a fast manner, and how these processes are integrated together to facilitate T-cell recognition.

Simultaneously, TCRs and other surface receptors are residing at the T-cell plasma membrane, which consists of a lipid bilayer with embedded proteins. It has been suggested that most plasma membrane-associated proteins are clustered in cholesterol-enriched “islands” (lipid rafts) that are separated by “protein-free” and cholesterol-low membrane [29, 36], shown in Figure 1. The composition of the membrane is regulated by lipid metabolism, which has been implicated in generating lipid rafts to facilitate T-cell function but the details remain unclear [125]. Epifluorescence, total internal reflection fluorescence (TIRF), and super-resolution microscopy experiments have together shown that TCRs form microclusters for effective antigen recognition and signaling [14, 29–33]. TCR microclusters are considered to be the signaling hotspots [32, 33], where the large CD45 protein tyrosine phosphatase molecules [126] are excluded to facilitate the binding and signaling of TCRs [32, 33, 61, 62, 113, 127]. The formation of TCR clusters is sensitive to the disruption of lipid rafts and the cytoskeleton [34, 36] and a recent study shows that cholesterol sulfate inhibits T-cell signaling [16]. We have also previously found that disruption of lipid rafts greatly reduced the 2D binding affinity of TCRs [11]. These studies together highlight the importance of the membrane lipid microenvironment in modulating cell surface receptor activation. However, the force regulation of lipid membrane signaling is not well studied. Recently, Xu and colleagues used single-molecule atomic force microscopy (AFM) to study conformational dynamics of the CD3ε cytoplasmic domain binding to the lipid plasma membrane and reveal multiple conformational states with different openness of three functional motifs [128]. This study suggests the fundamental importance of force in regulating lipid and T-cell signaling. To fully understand how mechanical forces regulate lipid rafts and TCR binding and signaling, more mechanistic studies combining force, metabolism, and signaling measurements are required to fully elucidate this interplay between forces and lipid structures at the T-cell membrane.

## T-CELL MECHANOTRANSDUCTION STRATEGIES

T cells live in a three-dimensional microenvironment in which they not only contribute to but also exert and respond to mechanical forces of varying magnitude, direction, and frequency [129]. Throughout their life cycles, T cells are exposed to a wide range of tissues with distinct mechanical microenvironments and subjected to varying hemodynamic forces in the lymphatic and blood circulation [67]. In addition to the external forces, internally generated forces such as actin cytoskeleton contraction and ligand-receptor catch bond have also been implicated in regulation of major T-cell functions as described in previous sections, and further depicted here [73, 111]. Here we review biomechanical regulation of T-cell functions, describe a cohort of bioengineering tools applicable to investigate these functions, and discuss several force sensors for quantification of physical forces exerted by T cells over multiple time and lengths scales.

### Bioengineering Strategies to Study External Mechanical Force Regulation on T-Cells Function

Application of fundamental engineering principles and recent advances in manufacturing biomechanics, materials sciences, and tissue engineering enable specific mechanical perturbations at the cellular and subcellular levels for mechanistic mechanobiology studies. Here we discuss a few bioengineering tools that model pathophysiological mechanical forces exerted by T cells at the cellular or subcellular levels for mechanistic investigation of T-cell biology.

#### Extracellular Matrix (ECM) Manipulation for Microenvironmental Mechanical Cues

**FIGURE 2 |:**
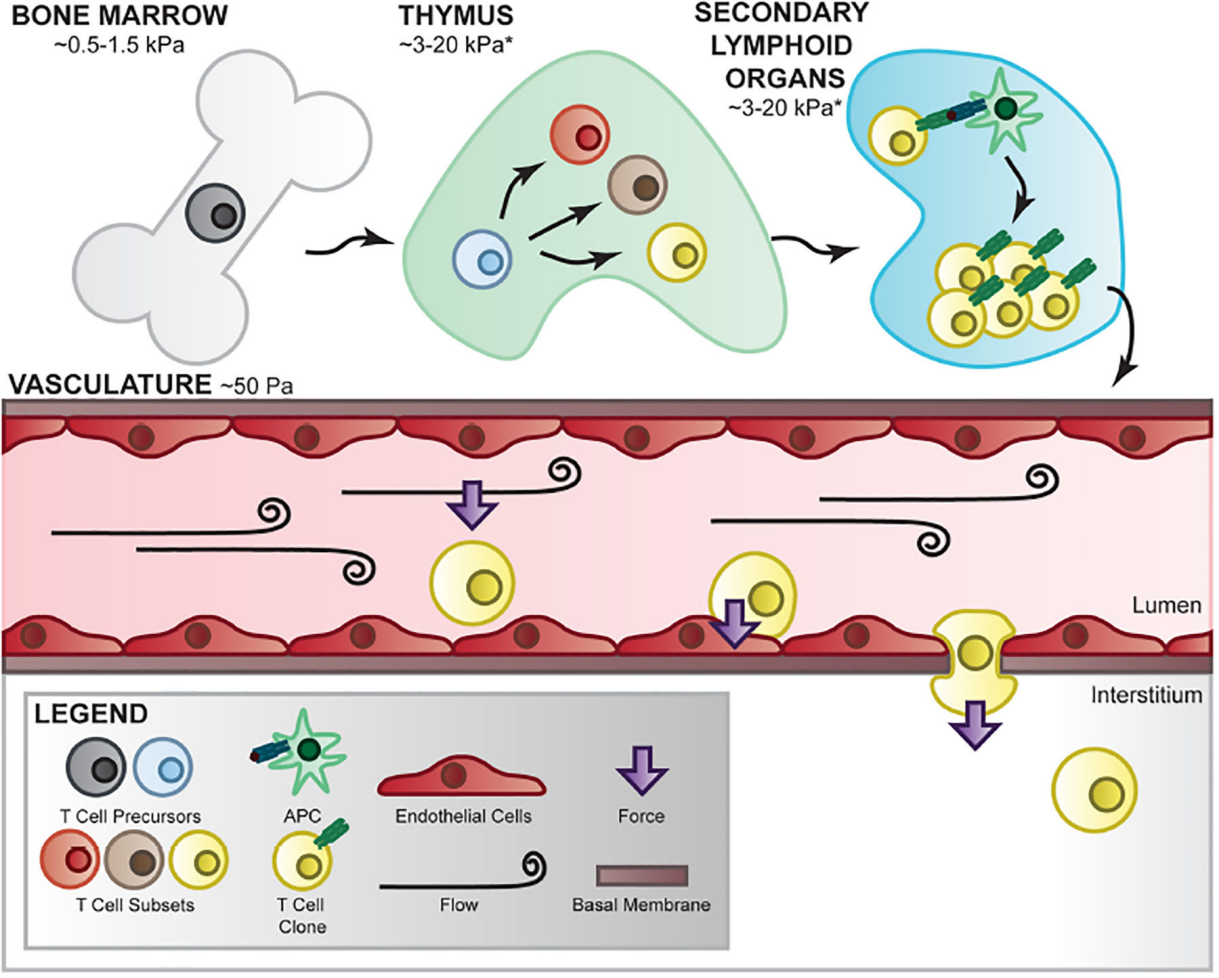
T-cell mechanical environment. T cells are subjected to various mechanical environments throughout their lifetime. During development and differentiation, T cells migrate between tissues of varying elasticity and extracellular matrix components which has been shown to affect their signaling and differentiation [130–132]. In the periphery, they are subjected to fluid flow-mediated forces which apply shear stress to the cells and their receptor/ligand interactions. In this environment, T cells are able to crawl along the vascular bed, adhere at the correct location, deform their shape, and propel themselves into the interstitial space to perform their immune function, all of which requires internally generated force as well as external mechanical regulation [133, 134]. *Estimation based on measured Young’s modulus on similar organs [135].

T cells encounter and function in a wide range of tissues comprising distinct cell populations and extracellular matrix (ECM) components through their development and during surveillance (Figure 2). Mechanical forces applied to and exerted by T cells are therefore dictated by the composition, architecture, and crosslinking of cell–extracellular matrix in these tissues with very distinct ECM elasticities. For instance, the Young’s modulus is estimated ~50 Pa in mucus and circulation, 0.5–1.5 kPa in bone marrow, 3–15 kPa in spleen, and 1,000–1,500 in cartilage [135]. It is important to note that the Young’s modulus is ~100,000 kPa in tissue culture plastic and ~70,000,000 kPa in rigid glass slides which are commonly used to culture T cells but do a poor job to recapitulate native microenvironmental mechanical cues. ECM mechanics are instrumental to cellular migration, growth, differentiation, morphogenesis, and tissue homeostasis [136], important biological processes intimately linked to T-cell functions and biology. Therefore, *in vitro* T-cell studies incorporating controlled matrix stiffness mimicking mechanical environments of tissues of interest may significantly strengthen the *in vivo* relevance of the findings. An array of biomaterials has been employed to engineer *in vitro* culture systems mimicking the *in vivo* mechanical properties of endogenous ECM mainly composed of elastic fibers, fibrillar collagens, glycosaminoglycans (GAGs), and proteoglycans (PGs). For instance, polyacrylamide hydrogels (in both 2D and 3D formats) have been widely used to engineer the microenvironments of variable stiffness for cellular studies in adhesion, differentiation, migration, proliferation, force generation, and cell-matrix interaction [130, 137, 138]. The elasticity of polyacrylamide hydrogels can be tuned precisely by altering the ratio of acrylamide monomer to the cross-linker of bis-acrylamide. Cellular responses to varying matrix stiffness from a few to hundreds of kPa have been investigated utilizing this tunable polyacrylamide hydrogel system. In addition to polyacrylamide, other materials such as Poly(dimethylsiloxane), Poly(ethylene glycol), alginate, and hyaluronic acid have also been utilized to engineer hydrogels with tunable elasticity for cell culture [139]. Using a 2D culture composed of poly(dimethylsiloxane)-based silicone elastomer, O’Connor et al. reported that *ex vivo* proliferation of human CD4^+^ and CD8^+^ T cells is significantly increased when cells are seeded in a substrates with Young’s modulus <100 kPa when compared to those on stiffer substrates with Young’s modulus >2 MPa [131]. In addition, the numbers of IFNγ-producing Th1 T cells are considerably increased when naïve CD4^+^ T cells are expanded on softer substrates (E<100 kPa) when compared with stiffer substrates (>2 MPa) [131]. Besides controlling mechanical properties of the tissues, ECM molecules connect to the cells through integrins, syndecans, and other receptors. Synthetic polymers with functional groups therefore are ideal to engineer hydrogels conjugating ECM proteins to study the biological consequences of different matrix protein–integrin pairs. Indeed, integrins on T cells not only bind to receptors on APCs and endothelium but also ECM proteins such as collagen, laminin, and fibronectin. For instance, fibronectin has been shown to co-stimulate T-cell proliferation via integrins a4b1 and a5b1 [132]. Nevertheless, the interplay between ECM elasticity and ECM protein composition in regulating T-cell action remains largely unexplored at the molecular level.

#### Flow Devices for Defined Hemodynamics

In the lymphatic and blood circulation as well as in the interstitial space, T cells are constantly exposed to hemodynamic forces generated by the flowing fluid, as shown in Figure 2. For instance, during immune surveillance naïve T cells dynamically circulate between the vasculature and lymph nodes where the interactions of fluid flow with local vessel geometry create complex hemodynamic characteristics including heterogeneous spatiotemporal shear stresses on the vessel wall. Hemodynamic shear stresses therefore not only govern major vascular functions but also play an important role in regulating critical T-cell functions such as crawling and extravasation (diapedesis) at the endothelial interface. Although underused in studying T-cell biology, an array of systems has been developed to apply well-defined hemodynamics investigating cellular responses to complex hemodynamic forces observed in the lymphatic and blood circulation as well as in interstitial space. For instance, parallel-plate flow chambers have been widely utilized to simulate fluid shear stresses on various cell types such as endothelial cells, smooth muscle cells, osteoblasts, osteocytes, cancer cells, and immune cells including leukocytes and T cells [133, 140–142]. In addition, cone-and-plate rheometers have been adapted to apply complex flow waveforms to cells that successfully elucidate novel mechanobiology insights related to cell morphology, gene expression, metabolic switch, and epigenome regulation [143–146]. A few studies using above-mentioned systems have provided lines of evidence supporting the importance of hemodynamic forces in regulating key T-cell functions. Steiner et al. employed a parallel flow chamber system showing T cells preferentially crawl against the direction of flow on the blood–brain barrier endothelial surface [133]. Using a cone-and-plate device, Schreiber et al. reported that hemodynamic shear stress promotes T-cell pseudopodial protrusions and consequent chemotaxis during lymphocyte extravasation from vascular endothelial cell monolayer [134]. Moreover, Woolf et al. reported using a parallel-plate flow chamber that shear flow interacts with CCL21 and integrin ligands to promote robust integrin-mediated adhesion of T cells to the endothelial monolayer [147]. However, molecular insights related to hemodynamic regulation of T-cell biology remain poorly understood. Recent advances in microfluidic devices that simultaneously model multiple mechanical cues (ECM sti ness and fluid flow) [148] could provide a powerful platform for future investigations of single T-cell responses to biophysical stimuli in a high-throughput fashion.

#### Methods to Study Force-Mediated Receptor-Ligand Interactions

T cells are able to sense and respond to external mechanical stimuli through a milieu of surface receptors, whose binding kinetics are mediated by force. For example, the TCR catch-bond forms selectively between an agonistic peptide but not an antagonistic peptide, described in section TCR-pMHC Catch Bond and depicted in Figure 1. Therefore, applying and measuring force to single surface molecules is critical to understanding how cells interpret and propagate mechanical signals to influence cell functions.

A common method for applying and measuring forces exerted by cells is Atomic Force Microscopy (AFM) [149]. In AFM, deflection of a spring-like cantilever scanned across a substrate can give topography of a sample at resolution on the order of fractions of a nanometer. As the tip of the cantilever comes into close proximity of the sample, the cantilever bends due to the force between the tip and sample. This deflection is monitored and is proportional to the force. Because the spring properties of the cantilever are known, and the tip can be tightly controlled spatially and temporally to make contact with the sample, AFM can be used not only as a means of imaging but also to both measure and apply forces at the few piconewton level. The tip can be functionalized to study receptor-ligand dynamics by adding a biotinylated ligand of interest to the streptavidin coated tip. This has been done to quantify adhesion forces and frequencies of the TCR/pMHC in both CD8^+^ [22] and CD4^+^[150] T cells. AFM can be coupled with fluorescence microscopy to allow for multiplexing fluorescent readouts such as calcium sensitive dyes, labeled receptors, or cytoskeletal rearrangement, enabling powerful real-time imaging.

Another method capable of studying single-molecule interactions is optical tweezers, which utilize the angular and linear momentum of light to manipulate microscopic objects [151, 152]. Optical traps are formed by tightly focusing an infrared laser beam to create a gradient of light intensity. Objects will be attracted to the point of highest intensity and “trapped” in three dimensions. The trapped object, normally a dielectric object such as a bead, will act as a Hookean spring where displacements can be correlated to force. Optical tweezers can be used to both measure or apply nanometer displacements corresponding to piconewton forces on single molecules attached to the bead, sometimes in the presence of an aspirated cell. For example, applying or measuring force at the TCR/pMHC interaction [27, 74, 79]. However, the use of isolated molecules functionalized to beads limits the physiological relevance of each measurement. Moreover, the beads can achieve angstrom spatial scale and second timescale but users have reported high experimental noise at longer timescales. Though the optical system allows for multiplexing other imaging modalities such as fluorescence or multiple optical traps, there are limitations to the amount of molecules that can be monitored at once which is a significant challenge for capturing the molecules’ native environment, especially in the context of T cells where multiple activation, co-stimulatory, checkpoint, and adhesion receptors cluster together and dynamically regulate each other’s kinetics.

The majority of force measuring methods utilize cells interacting with molecules functionalized to a bead, tip, or substrate. In order to encompass the complexity of a single molecules environment, methods that utilize cell-cell interactions can be used to monitor force such as the micropipette aspiration system [153]. In this system, single cells are held in place by aspiration into a micropipette. The pipettes can be driven by a piezoelectric translator to ensure contact of the two cells. Though one cell can be replaced by a bead, the strength of this system is being able to keep molecules within their semi-native environment to take real-time 2D kinetic measurements which has been shown to play an integral role in T-cell responsiveness [11, 154]. For TCR/pMHC interactions, an RBC is used as a surrogate APC to present pMHC, which is brought in and out of contact with a primary T cell aspirated by another micropipette with precisely controlled contact area and duration to yield an adhesion frequency. After generating adhesion frequencies over a range of contact durations, the binding affinity and off-rate are extracted using a monovalent binding kinetic model [153].

In another micropipette set-up, a functionalized probe bead can be attached to an aspirated cell and brought into close contact with another target bead. Named the biomembrane force probe (BFP), the RBC acts as an ultrasensitive force transducer, whose spring properties and deflection can be translated to sub-piconewton forces. For the thermal fluctuation assay [155], the RBC is attached by a pMHC-coated glass bead to form a biomembrane force probe, which is real-time tracked with nanometer spatial and sub-millisecond temporal resolution. The thermal fluctuation of the BFP is damped by the formation of a TCR-pMHC bond, allowing direct visualization of bond formation and dissociation and precise determination of bond lifetime in real time. The off-rate of single TCR-pMHC bond is extracted from the distribution of lifetime using a first-order irreversible dissociation model [11, 155]. A dual BFP has been developed to allow for multiple for temporally distinct ligand presentation to study crosstalk between surface receptors [156]. Finally, deformations of the aspirated cell morphology can be used to monitor pushing and pulling forces, which has been applied to studying T-cell responses to antibodies [157]. Similarly, to the aforementioned methods, any of these micropipette systems can incorporate fluorescent probes into the existing imaging system to monitor receptor clustering, and downstream signaling of binding events.

### Biophysical Force Sensors to Measure Internal Force Translation and Generation in T Cells

Recent biomechanical studies in cell biology have led to a cohort of force measurement systems that enable dynamic quantification of physical forces in cells on multiple scales from the cellular, subcellular, to molecular levels [158]. Here we review a few recently developed force sensors that serve as physical devices of known mechanical properties to receive biomechanical stimuli and transform them into measurable physical quantities. Particularly, force sensors that dynamically detect intracellular force changes due to cytoskeleton remodeling will certainly inform new molecular insights of T-cell biology given that T-cell trafficking and immunological synapse formation are actively regulated by the structure and function of cytoskeleton [73].

#### Traction Force Measured by Microscopy

Traction force microscopy (TFM) was developed to map and quantify the forces generated by cells against their substrates at the cellular and subcellular levels [159, 160]. Briefly, cells of interest are embedded in well-defined elastic hydrogel matrices (2D or 3D) that contain fluorescent microspheres. Traction forces exerted by the adherent cells are transmitted to the ECM via focal adhesions composed of structural and signaling molecules that form physical links between actin cytoskeleton and ECM. The deformation of the substrate, the result of cell contractile forces generated by the actomyosin cytoskeleton, can be detected by the displacement of fluorescent beads and quantified by elastic mechanics of matrices to yield vector maps of traction forces at the subcellular level. Notably, the substrate can be coated with varying ECM proteins to mimic the extracellular matrix of interest or with a ligand to study forces generated at the receptor-ligand interface [161]. TFM using ICAM-1–coated silicone-based gel was utilized to demonstrate that integrin-mediated force transmission in T cells requires actin-binding protein filamin A [162]. In addition to the continuous elastic substrates such as hydrogels, the elastic substrate can be microfabricated to form pillar arrays instead of a flat substrate so that each pillar acts as an independent cantilever [163]. TFM with micrometer-scale elastomer pillar arrays was used to show that T cells generate significant traction forces through TCRs and CD28 [76]. TFM can be easily integrated into existing fluorescent microscopy set-ups with modular cost and modification. However, TFM only reports cell-generated forces on an artificial substrate and is therefore not suitable for applying specific force to the cell or for studying cell-cell interactions.

#### Intramolecular Forces Measured by FRET

As described above, common methods for quantifying cellular forces can achieve physiological range but all either measure force generation onto extracellular substrates or apply known forces extracellularly to observe how cells respond. Since extracellular mechanical force is transmitted through surface receptors to the cytoskeleton and organelles, a method to measure internal forces between specific molecules is critical to understand the physiological relevance and mechanism of mechanosensitivity. In order to interrogate these mechanical interactions between single molecules at or beneath the cell surface, researchers have developed tension probes capable of entering cells that rely on the principles of FRET to reliably quantify force. FRET is commonly used to observe and quantify molecular interactions due to the distance dependent transfer of photons between two fluorophores. Because the efficiency of this transfer is dependent on proximity, changes in FRET efficiency can correspond to force-induced displacement of a small fluorophore labeled tensor [164]. One such tool utilizes DNA as the tension sensitive molecule to quantify TCR force transmission [165]. DNA is a popular tension sensor due to its simple synthesis, ease of fluorophore conjugation, and ability to tune the force required for unfolding at the piconewton level based on GC content and stem-loop structure [166, 167]. The FRET pair can also be connected via a flexible polypeptide linker to generate a completely genetically encoded tension sensor [168]. The sensor can be encoded between different molecules to measure intra-and inter-molecular tension such as between a and b integrin domains and between integrins and cytoskeleton within T-cells [65]. These DNA based molecular sensors allow for interrogation of single-molecule force transmission intracellularly which will be critical to understanding T-cell internal mechanobiology.

### TCR Signaling as a Result of Force

**FIGURE 3 |:**
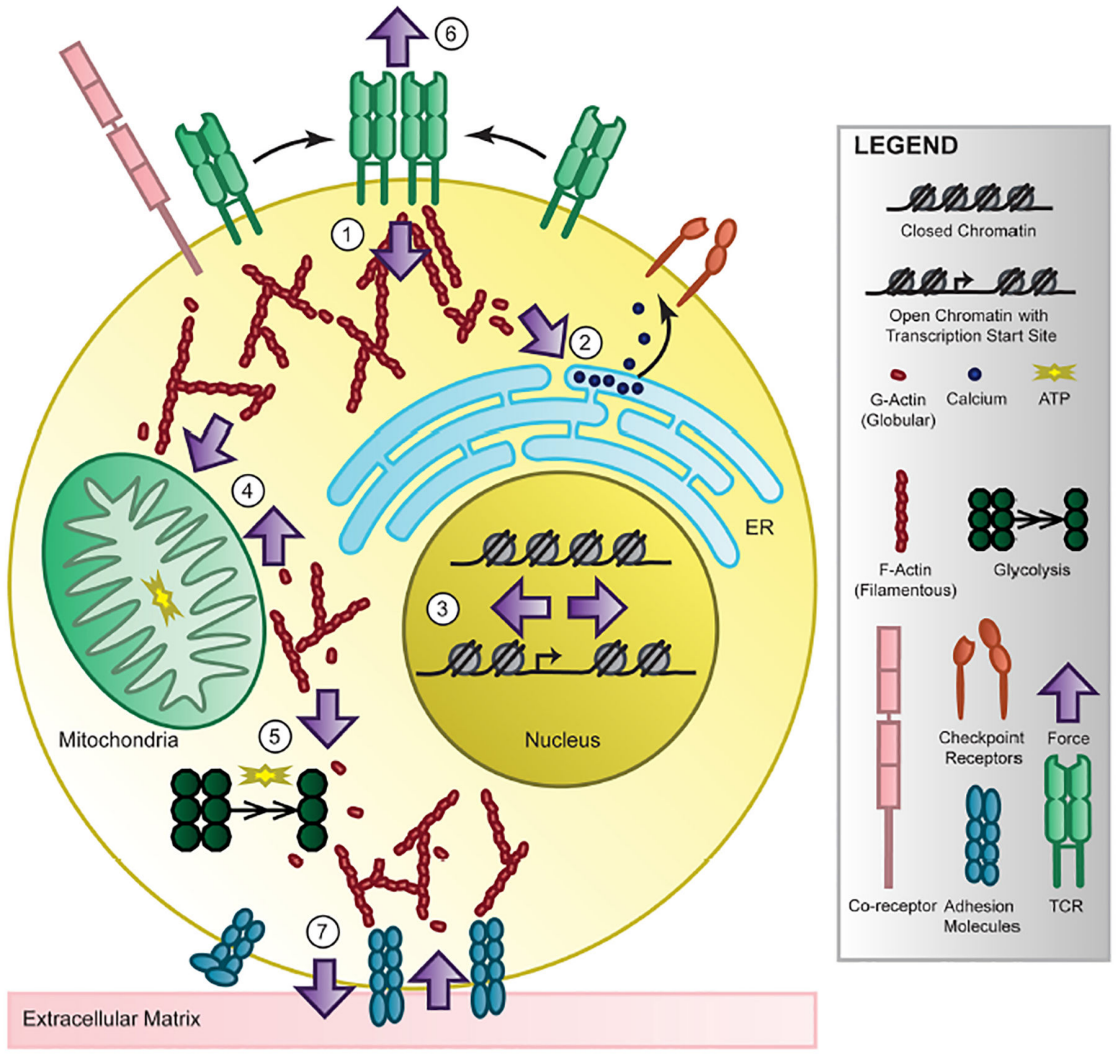
T-cell mechanotransduction. T cells perceive environmental force cues such as ligand binding, substrate stiffness, and shear stress through their surface receptors and membrane structure. These forces are internalized and transmitted at the cytoplasmic side of the receptors (1) and by the cytoskeleton, converted into biochemical signaling, then propagated to organelles such as the endoplasmic reticulum (ER) where calcium is released (2), the nucleus where the chromatin landscape and transcription changes (3), the mitochondria (4) or cytoskeletal-bound glycolytic compartments (5) to change energy production or back out to the surface of the T cell at the immunological synapse (6) or extracellular matrix (7) to modulate adhesion and cytokine release. Though accumulating evidence links force to T cell recognition, signaling, differentiation, metabolism, and genetics in T cells and others, the mechanisms require further study. However, energy-dependent cytoskeletal rearrangement is most likely the key to providing future investigators direction for uncovering the molecular players of T-cell mechanotransduction and elucidating force-dependent function.

Following force-mediated activation via the TCR, there are multiple signaling pathways that propagate throughout the cell to coordinate the effector function of the lymphocyte. Here we will focus on the major signaling pathways that follow T cell activation via the TCR, though they are coupled with costimulatory receptor and cytokine receptor signals, and describe the limited but promising evidence linking force to signaling. Receptor clustering following antigen recognition and activation at the immunological synapse localizes the internal signaling domains of the TCR co-receptors, CD4 and CD8, which bind the Src family protein tyrosine kinase Lck. Lck phosphorylates the immunoreceptor tyrosine-based activation motifs (ITAMs) on the CD3 cytoplasmic domains [169], which are able to recruit SH2-domain-containing proteins including ZAP70 to the TCR complex. Lck will also activate ZAP70, which will phosphorylate scaffolding transmembrane adaptor linker for activation of T cells (LAT) [170], creating docking sites for adaptor molecules which will recruit and activate multiple signaling molecules [171], serving as a activation signaling hub. For example, VAV1 activates RHO-family GTPases RAC and cell division control protein 42 (CDC42) to initiate actin polymerization and gene transcription. Cytoskeletal rearrangement is critical for synapse formation and cell motility but also can propagate force to other cellular components including the endoplasmic reticulum (ER), nucleus and mitochondria, possibly linking force at the synapse to T cell function, as depicted in Figure 3. Another LAT-docked signaling molecule is PLCg1 which will catalyze the production of second messengers to increase intracellular calcium by ER release and membrane channel opening, thereby activating calcineurin which will induce the nuclear localization of key transcription factor nuclear factor of activated T cells (NFAT). Both cytoskeleton rearrangement and intracellular calcium levels are critical to carrying out T cell function and a feedback mechanism between the two helps sustain this activation[172]. Interestingly, the translocation of the mitochondria close to the plasma membrane by the cytoskeleton can sustain calcium influx, thereby maintaining activation of T cells. Additional transcription factors including activator protein 1 (AP-1) and nuclear factor-κB (NF-κB) will become activated or localized to the nucleus by other signaling effectors, mitogen-activated protein (MAP) kinases and protein kinase C (PKC), collectively resulting in cell proliferation, cytokine production, and proliferation. Though TCR signaling has been extensively studied, how force regulates TCR signaling remains largely unknown.

Recent studies have started to assess canonical downstream T-cell signaling in the context of force. However, more studies need to be done to fully address how the mechanical environment and properties of T cells influence signaling and consequent function. The force-dependent TCR catch bond, described earlier in section TCR-pMHC Catch Bond, is required for triggering and sustaining calcium mobilization by prolonging the TCR-pMHC lifetime [26]. High calcium levels required early and rapid accumulation of TCR-pMHC bond lifetimes. High interaction forces between T cells and peptide ligands displayed on dendritic cells promoted calcium mobilization and IL-2 secretion, a key cytokine produced by activated T cells to induce proliferation [18]. Extracellular matrix stiffness has been shown to significantly influence key signaling pathways in T cells. Seeding on softer substrates (<100 kPa) has been shown to increase proliferation of both CD4^+^ and CD8^+^ T cells and activated effector Th1 T cells, or IFNγ-producing, increased after naïve CD4^+^ T cells were grown on softer substrates [131]. Similarly, naïve CD4^+^ T cells activation, measured by attachment and IL-2 secretion correlates with increasing the Young’s modulus of the substrate up to 200 kPa. Phosphorylation of signaling molecules including ZAP70 was also increased, indicating the mechanical environment is playing a role in the activation and downstream signaling of these cells [161]. In addition to the biomechanical cues provided by the extracellular matrix, T cells are constantly exposed and respond to the hemodynamic forces generated by the blood flow and interstitial fluid. For instance, fluid shear stress regulates transendothelial migration of T cells via cytoskeleton and Gi protein-mediated chemokine signaling [173] and modulates lymph node chemokine-induced T cell migration by integrin-dependent signaling [147]. Though the readouts of the key T cell mechanobiology studies reviewed here include calcium flux, signaling protein phosphorylation, and cytokine production, the links between purely mechanical stimuli and downstream signaling require further elucidation but most likely involve critical regulation and propagation by the cytoskeleton as accumulating evidence suggests [79, 111, 116, 117, 174]. Nevertheless, future studies should seek to define the force-mediated signaling pathway to determine how exactly external force is translated into biochemical energy to better understand T cell activation and function.

## FUTURE DIRECTIONS

The interplay between mechanical forces and cellular functions was first suggested in early twenty century by D’Arcy Thompson who postulated a possible contribution of physical forces in controlling the size and shape of living organisms [175]. How cells sense and transduce biomechanical forces into biological signaling has since become an intensely studied field and the frontier of current cell biology. Nevertheless, T-cell mechanoimmunology is still in its infancy and understanding how T cells actively respond to varying biomechanical cues could lead to not only novel molecular insights in mechanobiology but also fruitful discoveries to facilitate the emerging T cell-based therapies. Here we pose outstanding questions and likely directions of T-cell mechanobiology, depicted in Figure 3.

### Force in T-Cell Selection and Differentiation

Although the field of T-cell immunology has started to realize the importance of mechanical forces in T-cell activation, very little is known about whether and how mechanical forces regulate T-cell selection and differentiation. It is known that T-cell selection and differentiation are controlled by chromatin landscape and transcription factors in T-cell programming [176]. It is also known that mechanical forces can regulate chromatin structures: changes in the mechanical properties of the perinuclear cytoskeleton, nuclear lamina and chromatin are critical for cellular responses and adaptation to external mechanical cues. It has been found that altered nuclear mechanics are associated with many human diseases [177, 178]. However, it is poorly understood how forces regulate T-cell selection and differentiation.

For T-cell selection, the recognition of self-pMHCs on thymic APCs is critical for determining the fate of developing T cells. Somewhat paradoxically, recognition of self can elicit diametrically opposed outcomes. According to the classical affinity model, weak interaction is required for cell survival (positive selection) while strong interaction causes cell death (negative selection). However, the strength of binding affinity that determines negative and positive selection is poorly defined, mainly described as weak or strong interactions. It is not clear which affinity value/threshold can lead to positive selection or negative selection. It also does not take into account the fact that positive and negative selection largely occur in discrete thymic microenvironments, namely the cortex and the medulla, respectively. In addition to biological cues, both compartments have different force loads, thereby providing very different biomechanical microenvironments that orchestrate a spatial and temporal segregation of thymocyte selection [179, 180].

For T-cell differentiation, it is generally thought that the cytokine environment plays a central role in cell fate determination and effector function. The distinctive differentiated states of the various T-cell subpopulations are determined largely by the set of transcription factors they express and the genes they transcribe [181]. Although we know that T cells migrate and differentiate into distinct subpopulations at different organs of the human body, where they encounter different antigens and are subjected to different mechanical forces, the importance of TCR binding strength and the environmental mechanical cues are mainly ignored or understudied. It has been found that the matrix elasticity controls stem cell fate [130], and it is not known whether and how the TCR binding strength and forces regulate T-cell differentiation. We have known that binding of the TCR to different pMHCs exhibits different interaction characteristics in T-cell selection and activation. However, the role of TCR binding force in determine T-cell differentiation and memory is much less understood. For example, why the majority of effector cells undergo apoptosis but only a small subset (5%) of T cells change to a memory phenotype after the clearance of infections. Besides cytokines, do other environmental cues like force play a role in this process?

To fully understand the mechanisms of T-cell selection and differentiation, in addition to existing biological and chemical cues, we need to link the mechanical forces with traditional biochemical cues under physiological settings using precise quantitative analyses, instead of only qualitative descriptions.

### Force in T-Cell Metabolism and Genetics

In addition to aforementioned mechanotransduction mechanisms we discussed in this review, we believe that the integration of emerging research fields such as cellular metabolism and human genetics may significantly influence future T-cell mechanoimmunology investigations. Accumulating evidence strongly suggests the pervasive role of metabolism, the sum of biochemical reactions in living organisms that produce or consume energy, in regulating essentially every aspect of biological functions [182]. Core metabolic pathways such as glycolysis and oxidative phosphorylation (OXPHOS) have been long recognized to provide energy source for cells to sustain life. In addition to molecule degradation for energy release (catabolism), cellular metabolism is the major control for complex macromolecules synthesis and biomass creation (anabolism) which are instrumental to lymphocyte signaling and functions. For instance, naïve T-cells primarily rely on OXPHOS and exhibit low anabolic capacity given limited need for de novo synthesis of DNA, lipids, and proteins while integrated signals from pre-TCR, Notch1, and CXCR4 can temporarily increase glycolysis and promote anabolic metabolism during times of T-cell proliferation in thymus. Metabolic reprogramming is equally important during effector T-cell differentiation/activation. TCR ligation, co-stimulation, and cytokine signaling have been shown to collectively induce metabolic remodeling of naïve T-cells to promote anabolic growth and biomass accumulation [183] necessary for clonal expansion of antigen-specific T cells. Notably, phenotypic switch to activated effector T-cells requires not only elevated OXPHOS but also aerobic glycolysis (Warburg effect), a biological process in which glucose is converted into lactate even in the presence of normal levels of oxygen. It is thought that although OXPHOS (which produces 32 ATP per molecule of glucose) can sufficiently supply the ATP demands during T-cell activation, aerobic glycolysis (which produces only 2 ATP per molecule of glucose) is necessary for redox balance (NAD^+^/NADH) in the cell and the synthesis of metabolic intermediates important for cell growth and proliferation [184]. Moreover, emerging studies demonstrate that metabolic pathways and signal transduction are tightly regulated in a reciprocal fashion and strategic integration of metabolic and signaling cues allows cells to modulate key activities such as proliferation and differentiation depending on the metabolic status [185]. For instance, the cooperation between transcription factors and the chromatin landscape, important for the spatiotemporal control of gene expression programs and cell lineage determinations, are dynamically regulated by intermediate metabolites of cellular metabolic pathways. Acetyl-CoA and NAD^+^ generated from oxidative metabolism are key substrates for histone acetyltransferase (HAT) and histone deacetylase (HDAC) which control histone acetylation/deacetylation and consequent chromatin remodeling [186] crucial for regulation of cytokine gene expression during T-cell differentiation and activation. Whether and how mechanical forces regulate immune cell metabolism (“Mechanoimmunometabolism”) remains virtually unexplored but few lines of evidence from non-immune cells may provide some possible molecular insights. First, fluid flow shear stress has been recently reported to be a critical regulator of endothelial metabolic reprograming [146, 187]; unidirectional blood flow promotes endothelial OXPHOS while reduced shear stress increases glycolysis by stabilizing HIF-1a. Second, Notch1 that is necessary for T-cell lineage commitment was recently identified as a fluid shear stress sensor during vasculogenesis [188] and in adult artery [189]. At the molecular level, Hu et al. reported that actin cytoskeleton remodeling induced by small GTPase Rac1 directly regulates glycolytic outputs in epithelial cells by freeing the low-activity state, actin-bound aldolase A [124].

Recent human genetics studies not only significantly advanced our knowledge in the genetic basis of complex diseases associated with T-cell functions but also provide a new avenue for future T-cell mechanotransduction investigations. Human genetic mutations have been well-established as major regulators of T-cell homeostasis and dysfunctions related to a variety of diseases such as multiple sclerosis, autoimmune disease, and acute lymphoblastic leukemia [190–192]. Although the putative role of genetic variants in regulating T-cell mechanosensing biology remains to be elucidated, recent investigations have suggested that genetic predisposition provides a previously unappreciated layer of regulatory control in cellular mechanosensing mechanisms. For instance, mutations in DPP homolog 4 (SMAD4) in humans is hypothesized to promote epithelial b1-integrin mechanosignaling, leading to matricellular fibrosis, increased tissue tension, and tumor progress [193]. In addition, a genetic variant at phospholipid phosphatase 3 (PLPP3) implicated in coronary artery disease and ischemic stroke by genome-wide association studies (GWAS) has been shown to regulate endothelial mechanosensing responses to hemodynamic forces [194]. It remains poorly understood regarding the interplay between genetic predisposition and mechanosensing mechanisms converging on key T-cell functions, an area has tremendous potential to provide novel molecular insights in diagnosing and treating human genetic diseases associated with T-cell homeostasis and dysfunction.

### Concluding Remarks

Major efforts of mechanotransduction investigations have been focusing on identifying putative mechanosensors and cellular components in isolation. It remains poorly understood how the whole cell and entire tissue process and integrate this molecular scale information and further orchestrate physiologically relevant responses in the context of the multiscale architecture of our whole bodies. T cells serve as an ideal model system for an integrated approach to elucidate the dynamic interactions of individual components that operate at multiple spatiotemporal scales to mediate the cellular mechanosensing responses. Not only are T cells uniquely exposed to diverse external environments with distinct mechanical cues during their life cycle but major “–omics” techniques and systems biology approaches are becoming mature in T-cell investigations. These would allow investigators to monitor and record T-cell responses to mechanical perturbation at multiple levels of regulatory controls in a high-throughput fashion, providing novel molecular insights by which biological information flows from DNA to proteins to metabolites to cell structures to cell interactions in the context of T-cell mechanobiology.
